# Supplementary material for: Satellite repeat transcripts modulate heterochromatin condensates and safeguard chromosome stability in mouse embryonic stem cells
Source: Nat Commun. 2022 Jun 20;13:3525. doi: 10.1038/s41467-022-31198-3 (PMC9209518; doi:10.1038/s41467-022-31198-3)
Supplement: Supplementary file 2 — Description of additional Supplementary File [file 41467_2022_31198_MOESM2_ESM.pdf]

Descriptions of additional supplementary files

Supplementary Movie 1: Visualising chromocenters throughout the cell cycle, Related to Figure 1. Time lapse imaging of ESCs after doxycycline-induced expression of TALE-GFP against major satellite repeats (MSR). Images were acquired every 15 min over 72 time frames (18 hrs).

Supplementary Movie 2: Rapid movement of chromocenters, Related to Figure 1. Time lapse imaging (compiled five frames per second) of a representative ESC expressing doxycycline-induced TALE-MSRmClover. Images were acquired every 2 min for 60 frames (2h). Videos show cleavage and coalescence events with raw (a and c) or segmented (b and d) TALEMSR-mClover signal.

Supplementary Movie 3: Chromocenter dynamics, Related to Figure 1. Time-lapse imaging (compiled five frames per second) of an ESC cell expressing doxycycline-induced TALE-MSR-mClover, simultaneously showing the three coordinate axes (cross-sectional view) of chromocenter dynamics. Top-left panel: x-y; top-right panel: y-z and bottom-left panel: y-z. Images were acquired every 30 sec for 40 frames (20min).

Supplementary Movie 4: FRAP analysis of chromocenters, Related to Figure 1. Time lapse imaging (compiled five frames per second) of a representative ESC expressing doxycycline-induced TALE-MSRmClover and after GFP gapmer transfection. One chromocenter was photobleached and images acquired every second for 310 seconds.

Supplementary Movie 5: FRAP analysis after MSR RNA depletion, Related to Figure 2. Time lapse imaging (compiled five frames per second) of a representative ESC expressing doxycycline-induced TALE-MSRmClover and after MSR gapmer transfection. One chromocenter was photobleached and images acquired every second for 310 seconds.

Supplementary Movie 6: FRAP analysis of HP1α at chromocenters, Related to Figure 2. Time lapse imaging (compiled five frames per second) of a representative ESC expressing mEos3.2-HP1α and after GFP gapmer transfection.

Supplementary Movie 7: SIM 3D reconstruction of chromocenters, Related to Figure 3. Chromocenter 3D reconstructions acquired with structured illumination microscopy (SIM), showing details of H3K9me3 (green) and HP1α (red) at chromocenters in ESCs that were transfected with a) GFP or b) MSR gapmers (scale bars, 2μm).

Supplementary Movie 8: MSR RNA foci, Related to Figure 4. 3D video showing the nuclear location of MSR RNA foci (red) and chromocenters (blue) and their 3D models computed from segmented 3D images.
