# Supplementary material for: Satellite repeat transcripts modulate heterochromatin condensates and safeguard chromosome stability in mouse embryonic stem cells
Source: Nat Commun. 2022 Jun 20;13:3525. doi: 10.1038/s41467-022-31198-3 (PMC9209518; doi:10.1038/s41467-022-31198-3)
Supplement: Supplementary file 15 — Supplementary Figures [file 41467_2022_31198_MOESM15_ESM.pdf]

a)

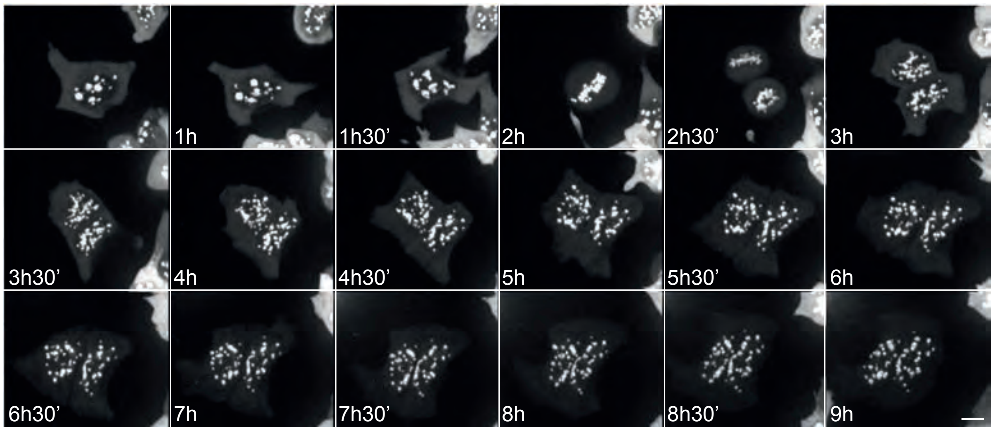
1h
1h30'
2h
2h30'
3h
3h30'
4h
4h30'
5h
5h30'
6h
6h30'
7h
7h30'
8h
8h30'
9h

b)

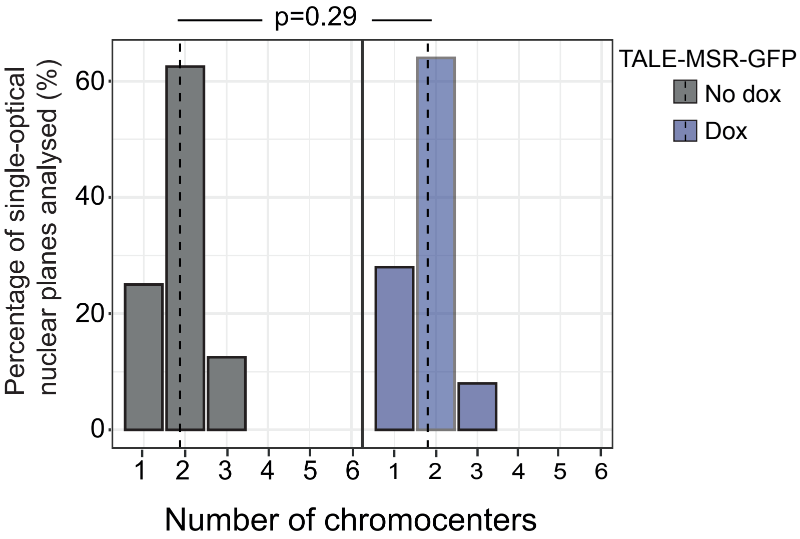
p=0.29
Percentage of single-optical nuclear planes analysed (%)
0
20
40
60
1 2 3 4 5 6 1 2 3 4 5 6
Number of chromocenters
TALE-MSR-GFP
No dox
Dox

c)

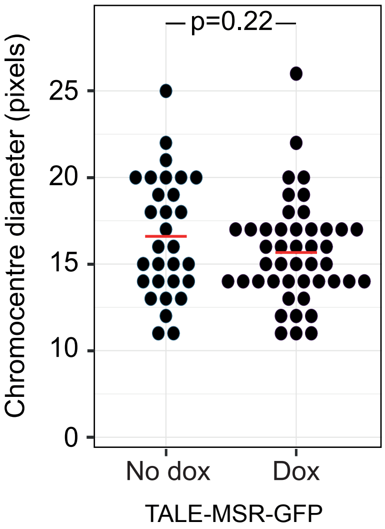
p=0.22
Chromocentre diameter (pixels)
0
10
15
20
25
No dox
Dox
TALE-MSR-GFP

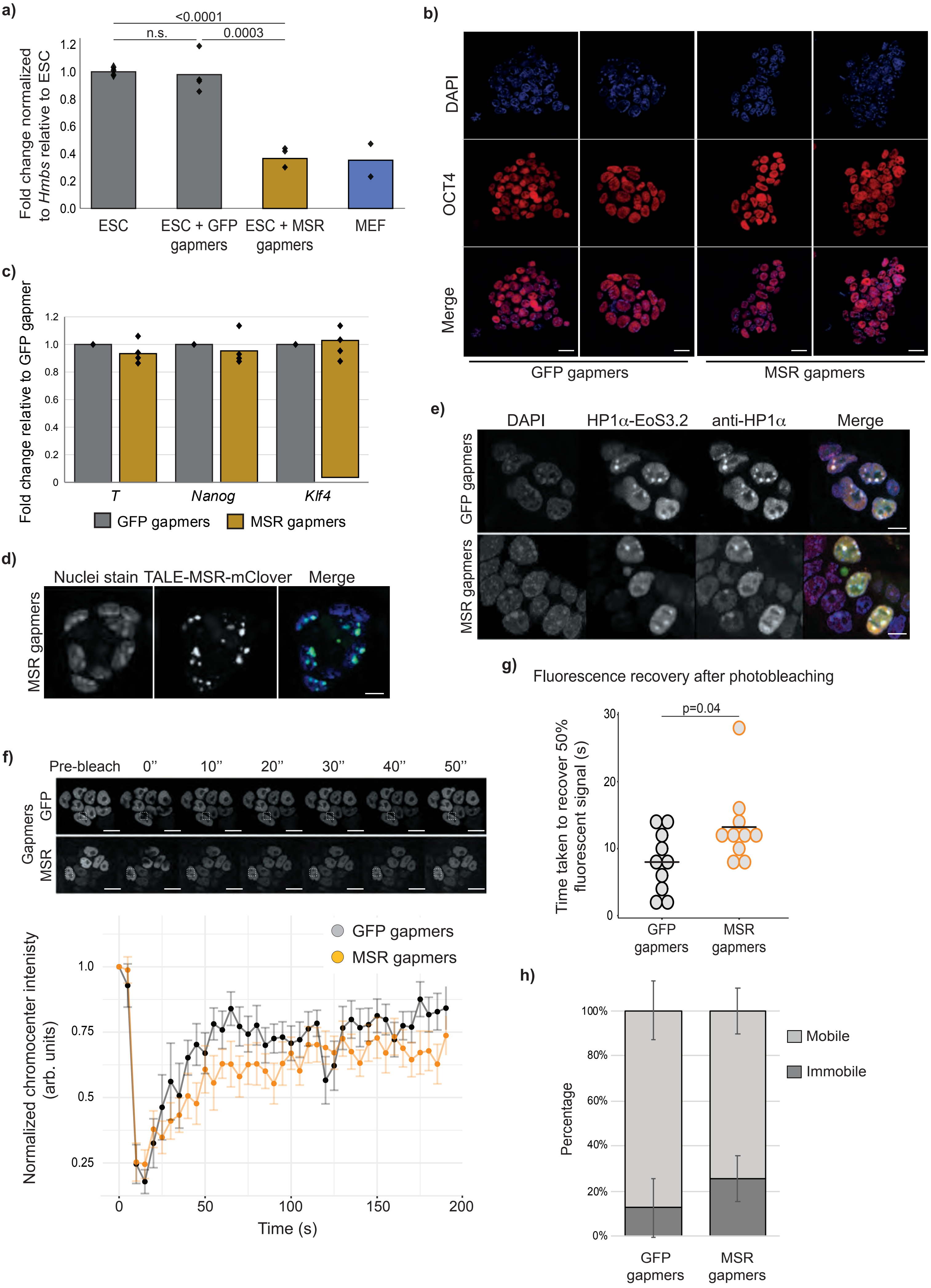
a)
<0.0001
n.s.
0.0003
Fold change normalized to Hmbs relative to ESC
1.2
1.0
0.8
0.6
0.4
0.2
0.0
ESC
ESC + GFP gapmers
ESC + MSR gapmers
MEF
b)
DAPI
OCT4
Merge
GFP gapmers
MSR gapmers
c)
Fold change relative to GFP gapmer
1.2
1.0
0.8
0.6
0.4
0.2
0
T
Nanog
Klf4
GFP gapmers
MSR gapmers
d)
Nuclei stain
TALE-MSR-mClover
Merge
MSR gapmers
e)
DAPI
HP1α-EoS3.2
anti-HP1α
Merge
GFP gapmers
MSR gapmers
f)
Pre-bleach
0''
10''
20''
30''
40''
50''
Gapmers
GFP
MSR
Normalized chromocenter intenisty (arb. units)
1.0
0.75
0.5
0.25
0
50
100
150
200
Time (s)
GFP gapmers
MSR gapmers
g)
Fluorescence recovery after photobleaching
Time taken to recover 50% fluorescent signal (s)
p=0.04
30
20
10
0
GFP gapmers
MSR gapmers
h)
Percentage
100%
80%
60%
40%
20%
0%
Mobile
Immobile
GFP gapmers
MSR gapmers

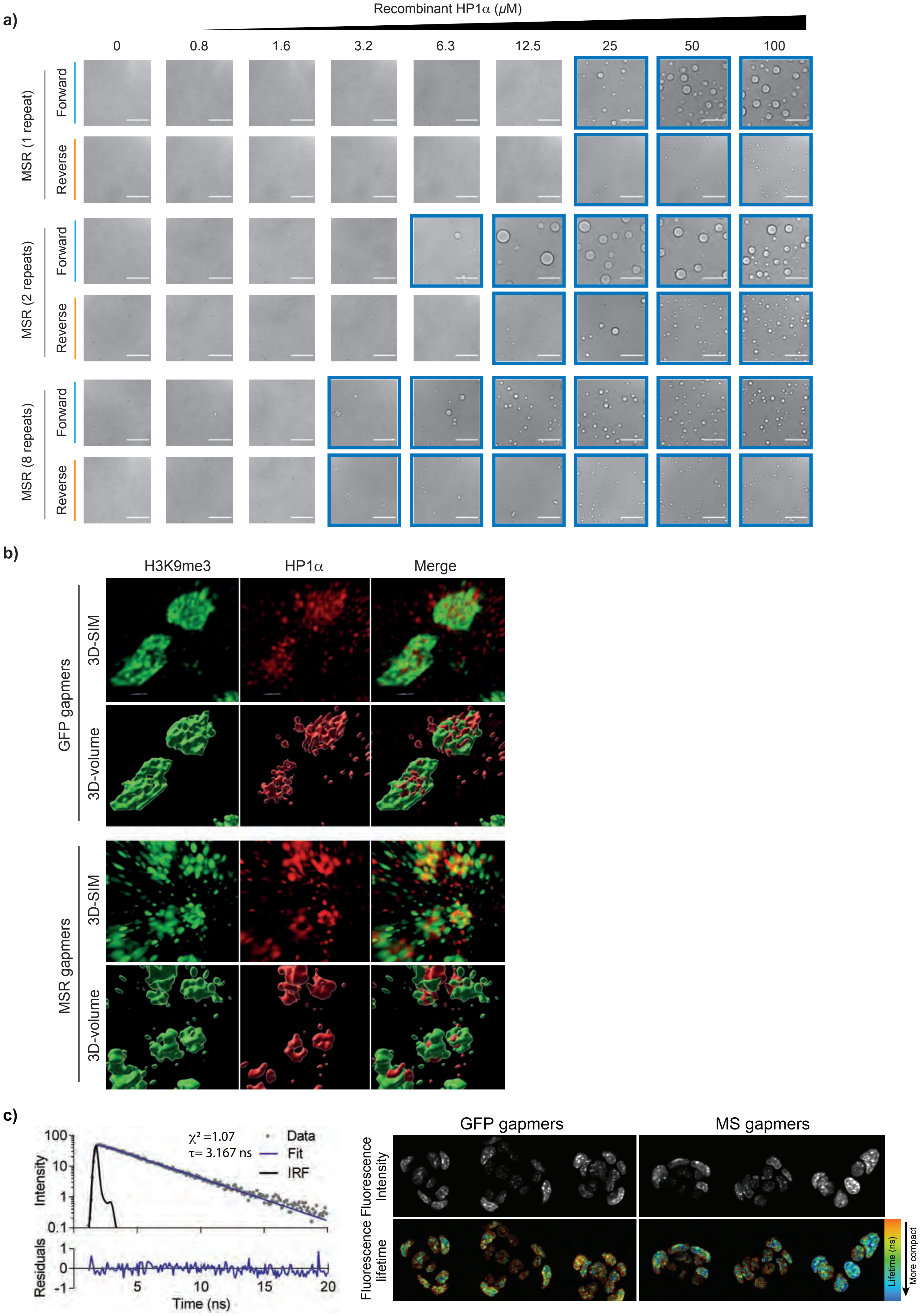
a)
Recombinant HP1α (μM)
0
0.8
1.6
3.2
6.3
12.5
25
50
100
MSR (1 repeat)
MSR (2 repeats)
MSR (8 repeats)
Forward
Reverse
b)
H3K9me3
HP1α
Merge
GFP gapmers
MSR gapmers
3D-SIM
3D-volume
c)
χ² =1.07
τ= 3.167 ns
Data
Fit
IRF
Intensity
Residuals
Time (ns)
GFP gapmers
MS gapmers
Fluorescence Intensity
Fluorescence lifetime
Lifetime (ns)
More compact

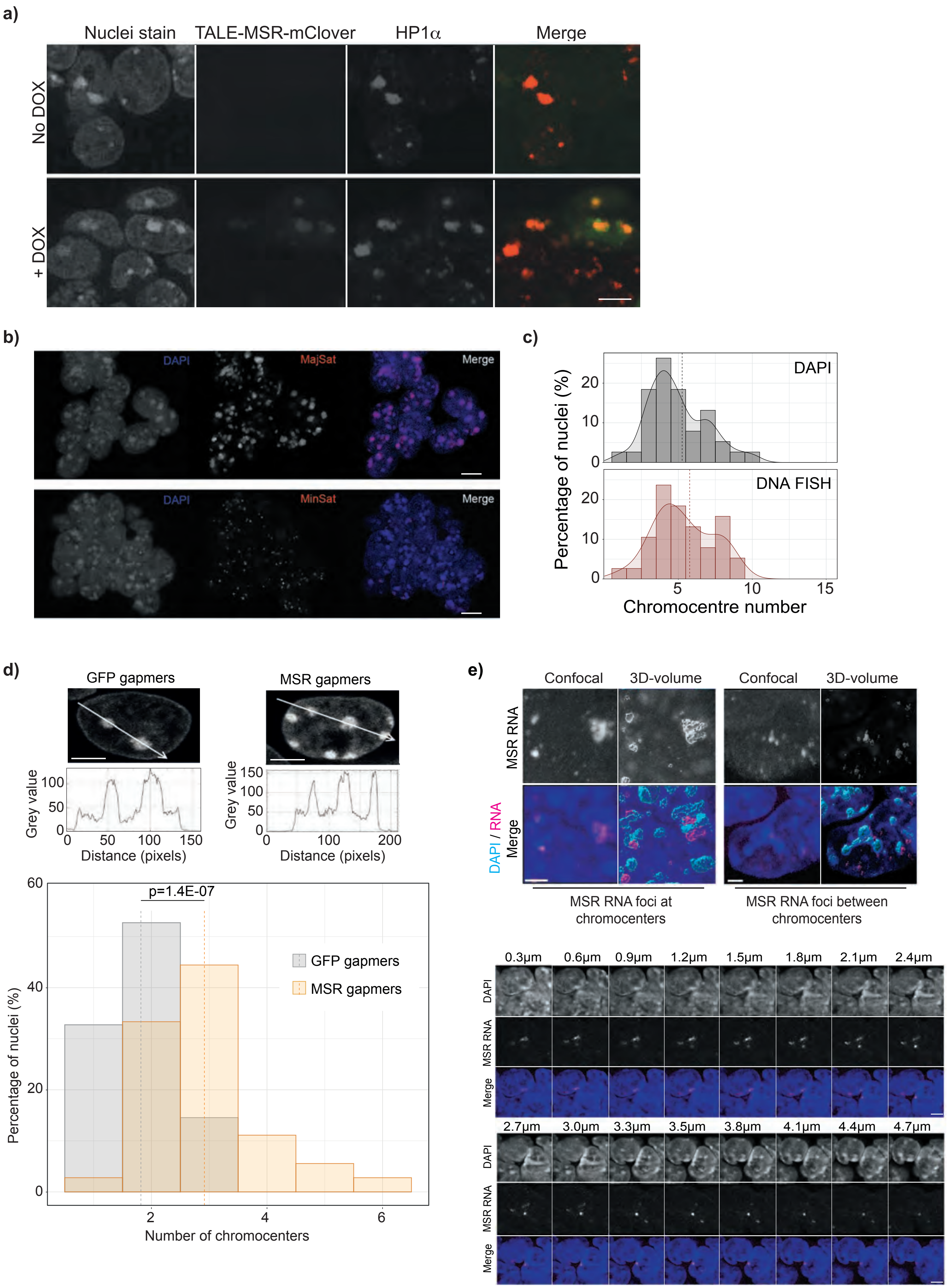

a)
Nuclei stain
TALE-MSR-mClover
HP1α
Merge
No DOX
+ DOX
b)
DAPI
MajSat
Merge
DAPI
MinSat
Merge
c)
DAPI
DNA FISH
Percentage of nuclei (%)
Chromocentre number
d)
GFP gapmers
MSR gapmers
Grey value
Distance (pixels)
Grey value
Distance (pixels)
p=1.4E-07
GFP gapmers
MSR gapmers
Percentage of nuclei (%)
Number of chromocenters
e)
Confocal
3D-volume
Confocal
3D-volume
MSR RNA
DAPI / RNA Merge
MSR RNA foci at chromocenters
MSR RNA foci between chromocenters
0.3μm 0.6μm 0.9μm 1.2μm 1.5μm 1.8μm 2.1μm 2.4μm
DAPI
MSR RNA
Merge
2.7μm 3.0μm 3.3μm 3.5μm 3.8μm 4.1μm 4.4μm 4.7μm
DAPI
MSR RNA
Merge

## Supplementary Figures

**Supplementary Fig. 1: Inducible targeting of GFP to satellite DNA with a TALE does not affect the nuclear organization of chromocenters, Related to Fig. 1.**

a) Images of ESC nuclei from a time-lapse experiment, which show that TALE-MSR-mClover binds to major satellite sequences throughout the cell cycle. Scale bar, 5μm.

b-c) Plots showing the number (b) and diameter (c) of chromocenter foci per ESC single focal plane across the mid-section of each nuclei, upon 24h doxycycline induction of TALE-MSR-mClover. This quantification was performed by analysing DAPI foci (chromocenters) of 16 nuclei (2D) with (DOX) or 25 nuclei without (no DOX) doxycycline induction of TALE-MSR-mClover. Data were collected from three independent experiments and compared using an unpaired two-sided Mann-Whitney test.

See also Supplementary Movies 1- 4.

**Supplementary Fig. 2: Embryonic stem cells do not differentiate when MSR transcript levels are reduced, Related to Fig. 2.**

a) Histogram of MSR transcripts levels in ESCs that were either untransfected or transfected with GFP or MSR gapmers, and in mouse embryonic fibroblasts (MEFs). Expression levels are shown as fold-change relative to *Hmbs*. Data collected from four independent biological replicates are shown and were compared using an unpaired two-sided Mann-Whitney test, except for MEF samples, where two replicates are shown.

b) Immunofluorescence microscopy images of OCT4 in ESCs, following transfection with GFP or MSR gapmers. DNA was counterstained with DAPI. Pluripotency markers where checked every time a replicate experiment was performed. N=3. Scale bars, 20μm.

c) Histogram showing the transcript levels of undifferentiated ESC marker genes (*Nanog*; *Klf4*) and differentiated cells (*T*), as measured by RT-qPCR. Data were acquired from three independent experiments and each dot represents individual biological replicates.

d) Representative images of live ESC nuclei showing TALE-MSR-mClover localisation at chromocenters is retained following transfection with MSR gapmers. N=3. Scale bar, 10μm.

e) Representative images showing the nuclear location of HP1α, sequentially detected by tagged HP1α-EoS3.2 (green), primary antibody (red) upon gapmer treatment. DNA is counterstained with DAPI (white). N=3. Scale bar, 10μm.

f) Top panel shows representative images of timelapse HP1α-mEos3.2 FRAP experiments in GFP or MSR gapmer-transfected ESCs. Dashed squares indicate the photo-bleached areas at 0s; note that the size of the bleached area was the same in all experiments. The lower panel shows GFP intensity (nuclear background and not-bleached chromocenter corrected mean intensity ± SEM) of photo-bleached chromocenters over

time after GFP or MSR gapmer transfection. Red dotted line shows the moment of photo-bleaching. A minimum of 30 cells per gapmer treatment were analysed.

g) Chart showing the increased time taken to recover 50% of the TALE-MSR-mClover signal at bleached chromocenters in ESCs with depleted MSR RNA levels following MSR-targeting gapmers. Each dot represents one photo-bleached chromocenter per photo-bleached acquisition (n=10 for each gapmer condition) and were compared using an unpaired two-sided Mann-Whitney test.

h) Histogram depicting the percentage ± SEM of the mobile and immobile components of chromocenters, as calculated from the FRAP data. Data were collected from different photo-bleaching experiments (n=10 for each gapmer condition).

See also Supplementary Movies 5-6.

**Supplementary Fig. 3: Satellite RNA promotes phase-separation of the heterochromatin protein HP1ɑ, Related to Fig. 3.**

a) Different lengths of forward MSR RNAs contribute to HP1ɑ droplet formation *in vitro*. Images framed in blue show the conditions that allow for the formation of HP1ɑ droplets. All images show the same magnification; scale bar, 100µm.

b) Representative 3D-SIM images after LNA-DNA gapmer transfection. For each experiment, the top panel shows the 3D-SIM reconstruction of H3K9me3 (green), HP1ɑ (red) and merged, and the lower panel shows the equivalent projected volumes.

c) Left panel: Typical fluorescence decay curve of SiR-DNA from a 5×5 pixel bin, with a monoexponential decay fitted using FLIMfit and fit residuals (error) plotted. The fluorescence decay curve is from a representative TCSPC-FLIM image of ESCs stained with SiR-DNA. IRF: Instrument Response Function. Right panel: Representative fluorescence intensity and lifetime images of SiR-DNA dye in ESCs transfected with control GFP and MSR gapmers.

See also Supplementary Movie 7.

**Supplementary Fig. 4: Characterisation of chromocenters following the depletion of major satellite RNA, Related to Fig. 4.**

a) Representative images of ESC nuclei with or without doxycycline-induction of TALE-MSR-mClover and counterstained for DAPI and HP1ɑ. In the merged image, HP1ɑ is shown in red, and TALE-MSR-mClover is shown in green. N=3, scale bars, 10µm.

b) Representative images of DAPI-stained ESC nuclei showing the distinct nuclear organization of major (top middle panel, MajSat-FISH) and minor satellite DNA (lower middle panel, MinSat-FISH). N=3, scale bar, 10µm.

c) Concordant distribution of chromocenter number per single-optical stack through whole nuclei that were quantified in 182 cells with either DAPI (top, grey) or MSR DNA-FISH signal (bottom, red). The dashed vertical lines show the mean chromocenter number for each signal.

d) Upper: Chromocenter organization as revealed by microscopy and linescan analysis of DAPI-large foci measured in single optical sections at optimal focal planes, after ESCs were transfected with either GFP or MSR gapmers. Scale bars, 5µm. Lower: Histogram shows the number of chromocenters, as defined by large DAPI-foci in single focal planes across the mid-section of each nuclei. A total of 100 and 105 chromocenters (55 and 36 nuclei) were quantified following the transfection of ESCs with GFP and MSR gapmers, respectively. Data were collected from three independent experiments and were compared using an unpaired two-sided Mann-Whitney test.

e) Top: representative RNA-FISH images in ESCs showing examples of MSR RNA foci distribution in relation to chromocenters. In the merged images, DAPI is shown in blue and the MSR-specific RNA-FISH signal in magenta. N=3, scale bar, 2µm. Bottom: Single-section images of GFP gapmer-treated ESCs showing consecutive z-stacks (0.3 µm steps) of two neighboring cells exemplifying MSR RNA foci within (upper row) and between (lower row) chromocenters. Scale bar, 5µm.

See also Supplementary Movie 8.
