# Supplementary material for: Satellite repeat transcripts modulate heterochromatin condensates and safeguard chromosome stability in mouse embryonic stem cells
Source: Nat Commun. 2022 Jun 20;13:3525. doi: 10.1038/s41467-022-31198-3 (PMC9209518; doi:10.1038/s41467-022-31198-3)
Supplement: Supplementary file 16 — Reporting Summary [file 41467_2022_31198_MOESM16_ESM.pdf]

Corresponding author(s): Clara Novo and Peter Rugg-Gunn

Last updated by author(s): May 6, 2022

Reporting Summary

Nature Research wishes to improve the reproducibility of the work that we publish. This form provides structure for consistency and transparency in reporting. For further information on Nature Research policies, see our Editorial Policies and the Editorial Policy Checklist.

Statistics

For all statistical analyses, confirm that the following items are present in the figure legend, table legend, main text, or Methods section.

n/a	Confirmed	
☐	☒	The exact sample size (n) for each experimental group/condition, given as a discrete number and unit of measurement
☐	☒	A statement on whether measurements were taken from distinct samples or whether the same sample was measured repeatedly
☐	☒	The statistical test(s) used AND whether they are one- or two-sided<br>*Only common tests should be described solely by name; describe more complex techniques in the Methods section.*
☒	☐	A description of all covariates tested
☐	☒	A description of any assumptions or corrections, such as tests of normality and adjustment for multiple comparisons
☐	☒	A full description of the statistical parameters including central tendency (e.g. means) or other basic estimates (e.g. regression coefficient) AND variation (e.g. standard deviation) or associated estimates of uncertainty (e.g. confidence intervals)
☐	☒	For null hypothesis testing, the test statistic (e.g. F, t, r) with confidence intervals, effect sizes, degrees of freedom and P value noted<br>*Give P values as exact values whenever suitable.*
☒	☐	For Bayesian analysis, information on the choice of priors and Markov chain Monte Carlo settings
☒	☐	For hierarchical and complex designs, identification of the appropriate level for tests and full reporting of outcomes
☐	☒	Estimates of effect sizes (e.g. Cohen's d, Pearson's r), indicating how they were calculated

Our web collection on statistics for biologists contains articles on many of the points above.

Software and code

Policy information about availability of computer code

Data collection	FRAP data was collected using TrackMate v3.8.0 (Tinevez 2017) and ImageJ (2.3.0/1.53f, Schneider 2012).<br>Volumes and intensities were collected using Imaris (v9.5.1, Bitplane, Oxford) and ImageJ (2.3.0/1.53f, Schneider 2012).
Data analysis	The following software was used for data analysis:<br>Imaris (v9.5.1)<br>ImageJ (2.3.0/1.53f, Schneider 2012)<br>CellProfiler (v4.2.0)<br>Graphpad Prism 7, GraphPad Software<br>RStudio (v1.2.5033)<br>TrackMate (v6.0.3 & v3.8.0)<br>FLIMfit v4.12.1

For manuscripts utilizing custom algorithms or software that are central to the research but not yet described in published literature, software must be made available to editors and reviewers. We strongly encourage code deposition in a community repository (e.g. GitHub). See the Nature Research guidelines for submitting code & software for further information.

Data

Policy information about availability of data

All manuscripts must include a data availability statement. This statement should provide the following information, where applicable:

- Accession codes, unique identifiers, or web links for publicly available datasets
- A list of figures that have associated raw data
- A description of any restrictions on data availability

The data that support the findings of this study are provided in the Supplementary Information and Source Data files.

Field-specific reporting

Please select the one below that is the best fit for your research. If you are not sure, read the appropriate sections before making your selection.

- [x] Life sciences
- [ ] Behavioural & social sciences
- [ ] Ecological, evolutionary & environmental sciences

For a reference copy of the document with all sections, see nature.com/documents/nr-reporting-summary-flat.pdf

Life sciences study design

All studies must disclose on these points even when the disclosure is negative.

Sample size	All sample sizes are indicated in the figure legends or in the manuscript. Samples sizes were not predetermined based on statistical methods. For each experiment, the sample size was chosen following pilot assays or standards in the field for each technique: at least duplicate experiments for ChIP; triplicate experiments for RT-qPCR; >100 data points for microscopy analysis.
Data exclusions	Outliers were identified using GraphPad Prism 7 software and excluded in the following experiments: In the FLIM data set, three nuclei with aberrant high or low signals in both the euchromatin and heterochromatin compartments were excluded. In the FRAP data set in Figure 2, one data point was identified as a statistical outlier and was therefore excluded.
Replication	All of the main experiments listed in the manuscript have been successfully replicated at least twice as independent experiments, with most derived from three biologically independent samples. The number of independent samples and experiments are provided in the figure legends.
Randomization	We report on in vitro experiments where randomization is not required. However, all experiments were done in a 'randomized' fashion where culture dishes containing the same cell population were chosen at random for experimental perturbation, for example gapmer treatment.
Blinding	The experiments were not blinded but repeated in at least three biological triplicates to confirm differences found.

Reporting for specific materials, systems and methods

We require information from authors about some types of materials, experimental systems and methods used in many studies. Here, indicate whether each material, system or method listed is relevant to your study. If you are not sure if a list item applies to your research, read the appropriate section before selecting a response.

Materials & experimental systems

n/a	Involved in the study	
	x	Antibodies
	x	Eukaryotic cell lines
x		Palaeontology and archaeology
x		Animals and other organisms
x		Human research participants
x		Clinical data
x		Dual use research of concern

Methods

n/a	Involved in the study	
x		ChIP-seq
x		Flow cytometry
x		MRI-based neuroimaging

Antibodies

Antibodies used	Rabbit H3K9me3 (IF), Active Motif, 1/400, Cat# 39161<br>Goat HP1α (ChIP & IF), Abcam, 1/200, Cat# ab77256<br>Rabbit GFP, (IF), Abcam, 1/400, Cat# ab290<br>Phospho-Histone H2A.X (Ser139) (IF), 1/200, Sigma, Cat# 05-636-I<br>Rabbit H3K9me3 (ChIP), Abcam, 1/200, Cat# ab8898<br>Mouse OCT3/4, (IF), Santa Cruz, 1/200, Cat# sc-5279

Goat Anti-Rabbit Alexa Fluor 488, (IF), Abcam, 1/500, Cat# ab150077
Donkey Anti-Goat Alexa Fluor® 488, (IF), Abcam, 1/500, Cat# ab150129
Donkey Anti-Goat Alexa Fluor 594, (IF), Abcam, 1/500, Cat# ab150132
Donkey Anti-Mouse Alexa Fluor® 594, (IF), Abcam, 1/500.Cat# ab150108
Donkey Anti-Rabbit Alexa Fluor® 647, (IF), Abcam, 1/500, Cat# ab150075

Validation

All antibodies were validated by the manufacturers for both species and application, as per statement in their websites.
Rabbit H3K9me3 (IF), Active Motif, 1/400, Cat# 39161 - https://www.activemotif.com/catalog/details/39161
Goat HP1α (ChIP & IF), Abcam, 1/200, Cat# ab77256 - https://www.abcam.com/hp1-alpha-antibody-ab77256.html
Rabbit GFP, (IF), Abcam, 1/400, Cat# ab290 - https://www.abcam.com/gfp-antibody-ab290.html
Phospho-Histone H2A.X (Ser139) (IF), 1/200, Sigma, Cat# 05-636-I - https://www.sigmaaldrich.com/GB/en/product/mm/05636?gclid=CjwKCAjw682TBhATEiwA9crl39UwIyeZ4-tZziFUEPdtZ84XD4-kP_GkYdBhliU-Emnvh4WEVzrwcBoCSKsQAvD_BwE
Rabbit H3K9me3 (ChIP), Abcam, 1/200, Cat# ab8898 - https://www.abcam.com/histone-h3-tri-methyl-k9-antibody-chip-grade-ab8898.html
Mouse OCT3/4, (IF), Santa Cruz, 1/200, Cat# sc-5279 - https://www.scbt.com/p/oct-3-4-antibody-c-10
Goat Anti-Rabbit Alexa Fluor 488, (IF), Abcam, 1/500, Cat# ab150077 - https://www.abcam.com/goat-rabbit-igg-hl-alexa-fluor-488-ab150077.html
Donkey Anti-Goat Alexa Fluor® 488, (IF), Abcam, 1/500, Cat# ab150129 - https://www.abcam.com/donkey-goat-igg-hl-alexa-fluor-488-ab150129.html
Donkey Anti-Goat Alexa Fluor 594, (IF), Abcam, 1/500, Cat# ab150132 - https://www.abcam.com/donkey-goat-igg-hl-alexa-fluor-594-ab150132.html
Donkey Anti-Mouse Alexa Fluor® 594, (IF), Abcam, 1/500.Cat# ab150108 - https://www.abcam.com/donkey-mouse-igg-hl-alexa-fluor-594-ab150108.html
Donkey Anti-Rabbit Alexa Fluor® 647, (IF), Abcam, 1/500, Cat# ab150075 - https://www.abcam.com/donkey-rabbit-igg-hl-alexa-fluor-647-ab150075.html

Eukaryotic cell lines

Policy information about cell lines

Cell line source(s)	E14Tg2a mouse ESCs were obtained from Janet Rossant, and originate from one of several embryonal stem cell (ES) lines developed by M. Hooper in 1987.
Authentication	Cells were validated by protein and transcript analysis.
Mycoplasma contamination	All cell lines tested negative for mycoplasma.
Commonly misidentified lines (See ICLAC register)	No commonly misidentified cell lines according to the International Cell Line Authentication Committee were used in this study.
